# On the Use of Viburnum Opulus (L.) in Dysmenorrhœa and Uterine Pain*Read before the Materia Medica Society, September 28, 1882

**Published:** 1882-12-20

**Authors:** A. M. E. Purdy


### ON THE USE OF VIBURNUM OPULUS (L.) IN DYSMENORRHŒA AND UTERINE PAIN.*

BY A. M. E. PURDY, M. D.

VIRBURNUM OPULUS, L.—NAT. ORD. CAPRIFOLIACEÆ.

The botanists describe this as a nearly smooth and upright shrub or small tree, rising from five to twelve feet in height; stems several from the same root, branched above; leaves three-lobed, three-veined, broadly wedge-shaped or truncate at base, broader than long; lobes divaricate, acuminate, crenately toothed on the sides, entire in the sinueses; petioles have two or more glands at the base, channeled above; flowers white or yellowish, reddish-white, in rayed, pedunculated cymes; marginal flowers large and sterile; inner flowers much smaller, and fertile. Fruit an ovoid, bright red, elliptic, one-sided drupe, very acid, ripens late, and remains upon the bush after the leaves have fallen. It resembles the common cranberry, and is sometimes substituted for it.

*History.*—Viburnum opulus is a handsome indigenous shrub, growing in low, rich lands, woods, and borders of fields, in the northern part of the United States, Canada, Europe, and North Asia, flowering in June, and presenting at this time a very showy appearance. The flowers are succeeded by red and very acid berries, resembling the common edible or low cranberry, and remain through the winter.

*Pharmacy and Chemistry.*—The bark is the medicinal part used. As met with in the shops, it is in thin, longitudinal curved pieces, from one-fourth of an inch to two or three inches in length, and from two to six lines in width, with a grayish epidermis, and whitish-yellow or reddish-yellow internal integument; it has no smell, and a peculiar, not unpleasant, bitterish pungent and astringent taste. It is frequently put up by the Shakers, when it is somewhat flattened from pressure. It has not been analyzed, but is known to contain valerianic acid. It yields its properties to water or diluted alcohol.

For medicinal purposes we procure the bark of the root, shrub, and its limbs (the fresh bark is preferable), and make a tincture with alcohol of 75 or 80 per cent. (Hale.)

This tincture should have a dark-red color, and a peculiar acid odor, very similar to the odor of valerian.

*Therapeutic Properties and Uses.*—"High cranberry-bark," says Hale, "is a powerful antispasmodic, and, in consequence of this property, it is more generally known among American practitioners by the name of cramp-bark. It is very effective in relaxing cramps and spasms of all kinds—as asthma, hysteria, cramps of the limbs and other parts in females, especially during pregnancy, and it is said to be highly beneficial to those who are subject to convulsions during pregnancy, or at the time of parturition, preventing the attacks entirely if used daily for the last two months

*Read before the Materia Medica Society, September 28, 1882

of gestation." In the treatment of spasmodic dysmenorrhœa, for which variety this remedy is specially indicated, Hale prescribes the tincture—a few drops three times a day for a week previous to the expected period. When the pains commence he gives it every half-hour, or every fifteen minutes if the pains are severe. He has found it equally useful for the severe false pains preceding normal labor, which often render the woman's life a torture for weeks ; and he says "it is of great value in after-pains, and should be given after every pain." Cramps in the abdomen and legs of pregnant women he was able to control very quickly by it, and claims it will prevent miscarriage if given before the membranes are injured, and when the pains are spasmodic and threatening.

The use of the *virburnum opulus* for the relief of dysmenorrhœa does not seem to have originated from professional sources. It has been in domestic use for a very long period as a remedy in the painful affections of women. Hale first gained his knowledge of the plant from its domestic use ; he first used it in the form of a weak infusion, then in drop-doses of the tincture, etc. Of its use in cases of neuralgic and spasmodic dysmenorrhœa, he has yet to meet with a single case where it has failed to cure, and states : "So confident have I been of its almost marvelous powers that I have taken pains to look up some old cases dismissed years ago as incurable, in order to test this remedy on them. In every instance thus far it has cured these old obstinate cases. Its sphere of action seems to cover nearly the same grounds as galvanism." The same writer predicts its usefulness in spasmodic conditions of all hollow muscular organs and their muscular connections. "Nor have I decided whether it acts on the muscular tissues *directly* or indirectly through the motor nerves. It may prove to be a spinal remedy after all."

Dr. Meyer, of Wilkesbarre, Pa., in his suggestive pamphlet on Specific Medication, says ; "Viburnum opulus, or high cranberry, and viburnum prunifolium, or black haw, seem to be antispasmodic, and to have a specific action upon the uterus. I have only used the first-named of these. My employment of it has convinced me that it is a uterine sedative, and often a remedy for neuralgic dysmenorrhœa, and for the commonly associated spinal irritation."

I have been in the habit of giving the concentrated tincture in five and ten-minium doses for these conditions, and also as a preventative of threatened abortion. I believe that in the majority of cases it has accomplished the object for which it was given.

From a paper by Dr. Charles E. Hall, published in the "Phila. Med. and Surg. Reporter" for June 22, 1878, I have made the following abstract of cases :

1. Colored woman, aged thirty-five. Never pregnant, general health good, works hard as a washerwoman.

Menstruation regular, but pain severe ; says she passes something which looks like shreds and patches of flesh. No vag. ex. ; could not rest from work. Has used various remedies, all of which had failed except opium.

Gave viburnum op. three times a day for three weeks before menstrual period, and every half-hour when period arrived.

Reported after four monthly periods; was taking the remedy regularly, suffering each month, but not taking opium, the pain not demanding it. She ceased taking the viburnum, and reported afterward that she had suffered as much as ever, and would not take any more of my medicine, as it did not cure unless she was always taking it. This patient afterward returned for the same remedy.

2. Miss A., aged twenty. From the commencement of menstrual life has suffered severely each month; pains spasmodic; general health good; is subject to hysterical attacks; has been using opium in some form each time, usually requiring three or four doses during the first twenty-four hours.

Gave viburnum three times a day during the whole interval, every half-hour at the period. At the next menstrual period had no pain; remedy continued, but limited to two weeks preceding the period. Five menstrual periods have passed without pain. The patient persists in continuing the medicine.

3. Mrs. —, aged twenty-two. Married two years, no children; had no pain at menses until upset from a boat while the period was present. Has since suffered each month. Viburnum used four months; has only slight uneasiness now each month; no medicine for several months.

4. Mrs. —, twenty-one years of age. Four years married; has great pain since marriage, never before. V. ex. reveals an elongated neck; very small cervical canal; cervix has been dilated with temporary benefit. General health poor; very thin, constipated, and in low spirits.

Used sponge-tents immediately preceding each period, and prescribed viburnum for three months. It is twelve months since treatment was suspended. Menstruation occurs without pain. General health greatly improved. The viburnum was continued two months after the local treatment was suspended.

5. Mrs. —, aged twenty-five. Since 1876, when an abortion on account of placenta prævia was produced on her, she has suffered very much with spasmodic pain each month. Viburnum, two weeks before expected time, gave first time great relief; took it again before the next period, and was entirely relieved.

Many other cases, says Dr. Hall, could be given, but these show that positive effects were produced by the drug; and he predicts cure when the pain is spasmodic and neuralgic, palliation when it is congestive or pseudo-membranous. He also used it in menorrhagia with intense crampy pain; also obtained speedy relief in cases of uterine colic.

The only other allusion in literature I have found was a brief statement on page 137 of Piffard's "Materia Medica and Therapeutics of the Skin." The writer states that dysmenorrhœa is sometimes promptly relieved by pulsatilla and viburnum opulus.

My own attention was called to the drug by the suggestion of Dr. Piffard, but in its early use no records were kept. The following recorded cases will show somewhat of its action.

The preparation used was similar to the abstracts of the new Pharmacopœa. The dose, five grains. The strength of this abstract is as follows: One grain of the abstract equals two minims of the tincture. The tincture used was made from one part of the fresh drug to two parts of alcohol.

CASE I.—Mrs. ——, aged twenty-six. Married one year. Suffering from cellulitis, which has been aggravated since married. Menstruation regular, usually lasting three or four days. As long as she can remember, has suffered at menstrual periods. The first day the pain is very severe, gradually diminishing until the close of the period.

*May 11th.*—Patient says she suffers dreadfully. Commenced taking five grains of the abstract two days before period three times a day; as soon as period commenced, every three hours; continued it every three hours for three days.

*June 9th.*—No pain whatever.

*July 7th.*—Very little pain, probably caused by a drive of twenty miles. Patient was absent from town, and wrote for more of the remedy, remarking she would not be without it.

*September 5th.*—Menstruates without pain.

This patient's appetite improved while taking the viburnum.

CASE II.—Madame ——, aged twenty-three. Widowed six years. Married at fourteen, before she had menstruated. Menstruated for the first time two months after marriage. In fourteen months after marriage was delivered of a healthy child. In twenty-two months a second child was born. After the birth of this child the husband died, the patient was up every day and night, and her menses did not return for eleven months, at which time they were accompanied with severe pain. Suffers at present with retroversion, two small fibroids, and leucorrhœa. Various measures and remedies had been used to relieve pain until May 28th, when she commenced the use of the viburnum (took only three doses at each period), which gave great relief; but the patient went out of the city, and suspended the use of the drug for one month, when the pain returned, with a dull, heavy feeling.

*September 19th.*—Has been menstruating two days, with a very great pain low down in the abdomen and back. Took viburnum twice, when all pain ceased, and she did not repeat the remedy.

CASE III.—Miss ——, aged eighteen. Always had pain at menstrual periods; has taken the viburnum for four months, three times a day three days before the period, and every three hours when the pain commenced. Says it is the first thing that has given her relief, as she now only suffers a little dull feeling.

CASE IV.—Miss ——, aged twenty-two, short and stout; first menstruated at thirteen; always regular; for days always had pain, which commences with first appearance of menstruation, is central, low down, and bearing down; frequently passes shreds. Commenced the use of the viburnum in June, 1881. I find this patient continued the use of the remedy for about six months, with great *relief at first, and no pain afterward*, suspending its use.

CASE V.—Miss ——, aged twenty-five, sister of last patient; menstruated first at twelve and a half; is regular, and the flow lasts two days; always had pain, which commences several days before the flow, but upon its appearance pain ceases; pain is central, low down, and also in the back. June, 1881, commenced taking the drug; at first was careless in its use, but, when she found her sister had received benefit, she obtained a fresh supply and renewed its use, taking it sometimes three times a day, sometimes every three hours, for two days; it always modified the pain, and many times cut it short; has not used it for several months, and has very little pain.

CASE VI.—Miss ——, aged twenty-nine, suffering from cellulitis and hæmatocele. Suffers terribly at menstruation, and moderately during the intervals. Has taken large quantities of opium. Commenced taking the viburnum last May, three times a day, since which time the pain has been much less severe, and no opium has been used.

I could recite many cases of the use of the virburnum opulus in uterine colic and the pain of pelvic cellulitis, but I do not wish to weary your patience. So many remedies and methods of treatment have been proposed from time to time for the treatment of dysmenorrhœa and uterine pain—the "Index Catalogue" of the National Medical Library devoting five of its closely printed columns to the subject—that I feel a hesitancy in suggesting another. But I am confident, with Hale and Meyer, that we have in this drug a powerful uterine sedative, and am satisfied—if preparations of the fresh drug, as insisted upon by Hall, be used—that many cases beyond the reach of any other therapeutic acid, except opium, will be relieved, and more positive results will follow than from the use of viburnum prunifolium.—*N. N. Med. Journal.*

